# Simultaneous quantitation and identification of intact Nandrolone phase II oxo‐metabolites based on derivatization and inject LC–MS/(HRMS) methodology

**DOI:** 10.1002/dta.3689

**Published:** 2024-04-06

**Authors:** Panagiotis Sakellariou, Polyxeni Kiousi, Michael Petrou, Yiannis S. Angelis

**Affiliations:** ^1^ Doping Control Laboratory of Athens, Institute of Biosciences and Applications National Centre for Scientific Research “Demokritos” Athens Greece; ^2^ Cyprus Anti‐Doping Authority (CyADA) Nicosia Cyprus

**Keywords:** doping control, Girard's reagent T derivatization, LC–MS/(MS), nandrolone, phase II metabolism

## Abstract

Α sensitive and selective derivatization and inject method for the quantification of intact nandrolone phase II oxo‐metabolites was developed and validated using liquid chromatography ‐ (tandem high resolution) mass spectrometry (LC–MS/(HRMS)). For the derivatization, Girard's reagent T (GRT) was used directly in natural urine samples and the analysis of the metabolites of interest was performed by direct injection into LC–MS/(HRMS) system operating in positive ionization mode. Derivatization enabled the efficient detection of nandrolone oxo‐metabolites, while at the same time producing intense product ions under collision‐induced dissociation (CID) conditions that are related to metabolites of the steroid backbone and not to the conjugated moieties. Glucuronide and sulfate metabolites of nandrolone were chromatographically resolved and quantified in the same run in the range of 1–100 ng mL^−1^, while at the same time structure identification could be performed for each metabolite. Full validation of the method was performed according to the World Anti‐Doping Agency (WADA) International Standard for Laboratories (ISL). Nandrolone oxo‐metabolites were quantified in two sets of urine samples, the first set consisted of real urine samples previously detected as negative and the second set consisted of urine samples collected from two excretion studies after nandrolone decanoate administration. The results for 19‐norandrosterone glucuronide (19‐NAG) and 19‐noretiocholanolone glucuronide (19‐NEG) were compared with those obtained by traditional gas chromatography ‐ (tandem) mass spectrometry (GC–MS/[MS]) method.

## Graphical abstract

Accurate and convenient analysis of the intact phase II oxo‐metabolites of nandrolone under the provisions of WADA's TD2023IDCR was accomplished with a dilute derivatization reagent (Girard's reagent T) and inject strategy.

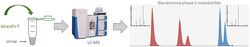

## 1 INTRODUCTION

Anabolic agents are used by athletes to enhance their performance, despite their prohibition by WADA and their well‐documented adverse health consequences.^1^, ^2^ 19‐norandrosterone (19‐NA) is the major metabolite of the prohibited anabolic steroid nandrolone or its prohormones and is considered in its glucuronidated form as the target metabolite in sports drug testing to reveal nandrolone abuse.^3^ Based on the 2021 Anti‐Doping Testing Figures, 62 Adverse Analytical Findings (AAFs) of 19‐NA were reported, in addition to 19 cases of Atypical Findings (ATFs) with the same detected substance, which warrants further investigations by the responsible testing authorities.^4^ All issues regarding the analysis and reporting of 19‐norsteroids related to nandrolone in the frame of sports drug testing are described in the mandatory to the WADA stakeholders TD2021NA.^3^ 19‐NA is among the substances whose simple detection is not sufficient to prove Anti‐Doping Rule Violation (ADVR), as it can also be produced endogenously in very low concentrations, and hence a urinary cut‐off concentration of 2.5 ng mL^−1^ is taken into consideration in Initial Testing Procedures (ITP) as alarm point leading to further analysis to uncover nandrolone abuse.^3^

However, above mentioned cut‐off concentration of 2.5 ng mL^−1^ can be exceeded not only after nandrolone administration but also in certain physiological cases that include: a) pregnancy,^5^, ^6^ b) consumption of wild boar offal,^7^, ^8^, ^9^, ^10^, ^11^ and c) rare cases of the so‐called “active urine” where demethylation of the endogenous C19 steroid, androsterone, converts it directly to 19‐NA.^12^ Additionally, the use of oral contraceptives based on the non‐prohibited substance norethisterone by female athletes produces 19‐NA in concentrations that exceed the cut‐off of 2.5 ng mL^−1^, but these findings are always accompanied by other norethisterone metabolites.^13^ Samples from male or non‐pregnant women athletes, not using norethisterone‐based contraceptives with 19‐NA concentration above the cut‐off of 15 ng mL^−1^ are reported as AAFs, without the need for further analysis.^3^ As an exception gas chromatography ‐ combustion ‐ isotope ratio mass spectrometry (GC‐c‐IRMS) analysis is provided for pregnant female athletes in any concentration of 19‐NA higher than 15 ng mL^−1.^ ^3^ In order to deal with cases a) to c) above, TD2021NA provides that further analysis of 19‐NA findings between the cut‐off levels of 2.5 and 15 ng mL^−1^ shall be performed with GC‐c‐IRMS, in order to discriminate the abuse of nandrolone preparations from cases where 19‐NA findings can be reasonably justified.^3^

Routine analytical procedures used by accredited doping control laboratories allow the determination of 19‐NA excreted free and conjugated with glucuronic acid, but amounts of 19‐nor steroids metabolites are known to be excreted in the sulfate fraction as well.^14^, ^15^, ^16^, ^17^, ^18^, ^19^ Sulfo‐conjugated nandrolone metabolites have been found to persist in human urine and the sulfo‐conjugated 19‐NA has been proposed as a long‐term marker for the enhancement of nandrolone abuse retrospective detection.^14^, ^15^, ^16^ However, the inclusion of the sulfate fraction as an additional target analyte(s) is not relevant in either ITP or confirmation procedures (CP) in the monitoring of nandrolone cases.^3^ There are two major reasons for this non‐inclusion of the sulfate fraction of steroids, such as nandrolone: the sub‐optimal sample preparation procedure available for their analysis and the prominence of the excreted glucuronide metabolites. Regarding the analysis of the sulfo‐conjugated metabolites, their inclusion in the traditional GC–MS/(MS) analysis of steroids requires their hydrolysis to their unconjugated counterparts prior to their analysis.^17^ Available options for this hydrolysis include either enzymatic hydrolysis or chemical hydrolysis. The advantages and pitfalls of this hydrolysis have been recently reviewed.^18^ Stereochemical and positional specificities and additional reactivities of aryl sulfatase enzymes, used to deliver this hydrolysis step, are problems of major concern.^19^, ^20^, ^21^ On the other hand, chemical hydrolysis can generate artifacts.^14^, ^21^

The direct analysis of the intact sulfate metabolites of nandrolone can be accomplished with other modern approaches like the liquid chromatography – (tandem) mass spectrometry (LC–MS/(MS)) in negative ionization mode.^15^, ^16^, ^22^, ^23^, ^24^ However, CID experiments produce product ion spectra with product ions derived exclusively from the sulfate moiety and hence lack of informative diagnostic product ion fragments that could be correlated with the steroid molecules.^23^, ^24^ In that respect LC–MS/(MS) analysis of sulfated metabolites cannot be used for the unambiguous identification of prohibited substances under the provisions of WADA Technical Document TD2023IDCR.^25^ The recent promising analysis of intact sulfates by GC–MS/(MS) as de‐sulfated artifacts^26^, ^27^, ^28^ generates many positional isomers for some sulfated steroids. Additionally, this methodology cannot be used to distinguish between stereochemical isomers in which sulfate group is located in the same carbon of the steroid molecule like in the cases of testosterone sulfate and epitestosterone sulfate^26^ or 19‐norandrosterone sulfate (19‐NAS) and 19‐norepiandrosterone sulfate (19‐NEAS) (data non show), because they produce the same de‐sulfated artifacts.

The development of a reliable and convenient methodology that would allow the inclusion of the sulfate fraction in the confirmation of 19‐NA findings would be valuable as a) it can prolong detection windows for nandrolone abuse,^14^, ^15^, ^16^ b) the 19‐NAS/19‐NAG ratio could be a candidate discrimination tool, complementary to the GC‐c‐IRMS, of tail nandrolone doping cases from other situations like pregnancy and consumption of wild boar offal as different excretion rates have been described for the glucuronide and the sulfate fraction of nandrolone related formulations in comparison with these cases.^22^ Toward this scope, a confirmatory procedure based on chemical derivatization with GRT^29^, ^30^ was developed for the quantitation and confirmation of the intact phase II nandrolone oxo‐metabolites in their glucuronide and sulfate forms by LC–MS/(HRMS) analysis in one run. Full validation of the assay for 19‐NAG, 19‐NEG, 19‐NAS, 19‐NEAS, and 19‐noretiocholanone sulfate (19‐NES) was performed according to WADA ISL.^31^ The validated method was further applied to real urine samples from administration studies, in order to confirm the effectiveness and sensitivity of the method, and the findings were compared with those obtained by the analysis of the same samples by the traditional GC–MS/(MS) methodology.

## 2 EXPERIMENTAL

### 2.1 Chemicals, reagents, and materials

Reference substances 19‐NA, 19‐noretiocholanolone (19‐NE), 19‐NAG, 19‐NEG, 19‐NAS, D4‐19‐norandrosterone glucuronide (D4‐19‐NAG), and D4‐19‐norandrosterone sulfate (D4‐19‐NAS), were purchased from National Measurement Institute (NMI, Canberra, Australia), whereas 19‐NES, 19‐norepiandrosterone (19‐NEA), and 19 norepietiocholanolone (19‐NEE) were purchased by Steraloids Inc. (Newport USA). Stock solutions of the reference substances were prepared in methanol and stored at −20°C. Ethyl acetate, n‐pentane, and methanol were of analytical grade and obtained from Labscan (Dublin, Ireland). β‐glucuronidase from *E. coli* was obtained from Roche (Basel, Switzerland). Dipotassium hydrogen phosphate (K_2_HPO_4_), potassium dihydrogen phosphate (KH_2_PO_4_), disodium carbonate (Na_2_CO_3_), sodium hydrogen carbonate (NaHCO_3_), sodium sulfate (Na_2_SO_4_), potassium carbonate (K_2_CO_3_) glacial acetic acid and acetonitrile of liquid chromatography ‐ tandem mass spectrometry (LC–MS/MS) grade were purchased from Panreac (Barcelona, Spain). Diethyl ether of analytical grade, formic acid for LC–MS/MS, sulfur trioxide pyridine complex, N,N‐dimethyl formamide (DMF), 1,4‐dioxane, sodium hydroxide (NaOH), ammonium formate for LC–MS/MS, Girard's Reagent T, ammonium iodide (NH_4_I), and 2‐propanethiol (2‐PrSH) were purchased from Sigma‐Aldrich (Steinheim, Germany). N‐methyl‐N‐trimethylsilyltrifluoroacetamide (MSTFA) was purchased from Chemische Fabrik Karl Bucher (Waldstetten, Germany). Milli‐Q water was produced in‐lab by a Milli‐Q purifying system (Millipore, Billerica, MA, USA)

For the synthesis of steroid sulfate metabolites, a previously reported method was employed.^32^ As a steroid reference solution that could be used for semi‐quantitative determinations would be highly desirable, we followed the process described below in order to have an indication of the concentration of the resulting products. More specifically the reaction was performed in duplicate under identical conditions. Aliquots from the first reaction mixture were taken at different time points and analyzed by LC–MS/(HRMS) after derivatization, in order to estimate the progress of the reaction. When reactants disappeared from the reaction mixture, a full workup was performed on the second reaction mixture where the final product was isolated.

In more detail, each one of 100 μl of the 19‐NEA and 100 μl of 19‐NEE solutions (1,000 μg mL^−1^ in methanol) was transferred into two different test tubes and evaporated to dryness under a nitrogen stream at 50°C. A total of 100 μl of 1,4‐dioxane was added to each test tube, and the mixture was vortexed for 30 sec. Following this, 100 mg of sulfur trioxide pyridine complex was dissolved in 200 μl of anhydrous DMF and added to the test tube. The mixture was stirred at room temperature. Aliquots of 5 μl were removed from the first test tube at different time points and each aliquot was transferred to a vial with an insert. A total of 10 μl of 0.1 M K_2_CO_3_ were added, and the mixture was diluted with 200 μl of 1 M GRT in 0.1 M acetic acid. The vial was heated at 70 ^ο^C for 20 min. After cooling, 5 μl of the mixture was injected directly into the LC–MS/(HRMS) system, where the molecular ions of the sulfated and the unreacted free steroids were recorded. When unreacted free steroids disappeared, the reaction mixture in the second test tube was diluted with 2 ml of Milli‐Q water to quench the reaction. The sulfated steroids were extracted with 5 ml methanol after solid phase extraction with preconditioned C18 cartridges, and the resulting methanolic solutions were used as stock solutions for further use.

### 2.2 Sample preparation

#### 2.2.1 Detection of nandrolone phase II oxo‐metabolites by LC–MS/(HRMS)

For the detection of nandrolone phase II oxo‐metabolites, 100 μl of urine were transferred to a 1.5 ml tube, and 20 μl of a mixture of internal standard (ISTDs) containing D4–19‐NAG and D4–19‐NAS (100 ng mL^−1^) were added. A total of 100 μl of 1 M GRT solution in 0.1 M glacial acetic acid was added, and the mixture was centrifuged for 10 min at 10.000 rpm. A total of 150 μl were transferred to a vial with an insert. The vial was heated in an oven at 70°C for 20 min and then cooled to room temperature. 20 μl of the sample was injected in LC–MS/(HRMS) without any further purification.

#### 2.2.2 Detection of nandrolone‐free and glucuronide metabolites by GC–MS/(MS)

For the extraction of free and glucuronide metabolites, D4–19‐NAG (50 μl from a solution of 0.3 μg mL^−1^) was added to 3 ml of urine as ISTD. pH was adjusted to 7.0 using phosphate buffer 1 M and urine samples were incubated for 1.5 h at 55°C, after the addition of 30 μl β‐glucuronidase from *E. Coli*. Following hydrolysis, urine pH was adjusted to 9.5 with NaHCO_3_:Na_2_CO_3_ (10:1, w/w) solid buffer and glucuronide‐hydrolyzed steroids were extracted with 5 ml n‐pentane. Samples were centrifuged for 10 min at 2.000 rpm and then the organic layer was transferred to the tubes and evaporated to dryness under a nitrogen stream at 60°C. Per TMS derivatives were prepared by adding 50 μl of MSTFA/NH_4_I/2‐PrSH (1,000:4:3, v/w/v) to the dry residue. The mixture was incubated at 80°C for 30 min and 3 μl of the sample was then injected directly into GC–MS/(MS).

### 2.3 Instrumentation

#### 2.3.1 LC–MS/(HRMS)

A Dionex UHPLC system (Thermo Scientific, Bremen, Germany) was used for the chromatographic separation. The system consisted of a vacuum degasser, a high‐pressure binary pump, an autosampler with a temperature‐controlled sample tray set at 15°C, and a column oven set at 30°C. Chromatographic separation was performed at 30°C using a Hypersil Green PAH column (100 x 2.1 mm i.d., 3 μm particle size; Thermo Scientific) connected to a Hypersil GOLD C18 column (50 x 2.1 mm i.d., 1.9 μm particle size; Thermo Scientific). The mobile phase consisted of 5mM ammonium formate in 0.02% formic acid (Solvent A) and a mixture of acetonitrile: water (90:10, v/v) containing 5mM ammonium formate and 0.02% formic acid (Solvent B). A gradient elution program was employed with Solvent B starting at 19% at a flow rate of 0.2 ml min^−1^, initially increasing to 20% in 10 min at a flow rate of 0.25 ml min^−1^ and then increasing to 21% in 27 min and to 25% in 36 min at a constant flow rate of 0.25 ml min^−1^. Then, Solvent B continued to increase to 100% in 40 min at a flow rate of 0.3 ml min^−1^ where it was held in 47 min before returning to 21 in 49 min at a flow rate of 0.2 ml min^−1^ where it was held in 55 min. The injection volume was 20 μl. The Diverter valve was programmed to send LC eluent in waste for the first 4.5 min.

A QExactive benchtop Orbitrap‐based mass spectrometer (ThermoScientific, Bremen, Germany) operated in the positive polarity mode equipped with a heated electro‐spray ionization (HESI) source was used as detector. Source parameters were: sheath gas (nitrogen) flow rate, auxiliary gas (nitrogen) flow rate, and sweep gas flow rate: 40, 10, and 1 arbitrary units, respectively, capillary temperature: 300°C, ESI heater temperature: 300°C, spray voltage: +4.0 kV (positive polarity). The instrument operated in full scan mode from *m/z* 50–750 at 17,500 FWHM resolving power and injection time of 200 ms and in PRM mode with an isolation window of 1.0 *m/z* and initial mass of 150.0 *m/z* at 17,500 FWHM resolving power and injection time of 100 ms (product ion mode). The automatic gain control (AGC) was set at 3e6 ions. The mass calibration of the Orbitrap instrument was evaluated in both positive and negative modes weekly and external calibration was performed prior to use following the manufacturer's calibration protocol.

#### 2.3.2 GC–MS/(MS)

A Trace 1,610 GC system (Thermo Scientific, Bremen, Germany), combined with a TSQ9610 (Thermo Scientific, Bremen, Germany), triple quadrupole mass selective detector equipped with an advanced electron ionization source was used. The system was equipped with an SGE BPX5 column (30 m length, 0.25 mm ID, 0.1 μm film thickness). Helium was used as carrier gas at a constant flow rate of 1.65 ml min^−1^. A total of 2 μl of the sample was injected in split mode (10:1). The initial oven temperature was set at 160°C, ramped at 10°C min^−1^ to 200°C, then ramped at 2°C min^−1^ to 220°C, at 6°C min^−1^ to 263°C and finally at 50°C min^−1^ to 310°C (held for 1.6 min). Argon was used as collision gas at a pressure of 4 bar. The MS is programmed in multiple reaction monitoring (MRM) modes.

### 2.4 Assay evaluation

Due to the fact that according to WADA TD2021NA important management decisions regarding nandrolone‐related findings are based on the concentration of 19‐NA,^3^ a quantitative assay was developed. This assay was targeting primarily the 19‐NAG considering the two cut‐offs referred to the TD2021NA, namely the cut‐offs of 2.5 and 15 ng mL^−1^, as thresholds.^3^ This was found necessary as the GRT derivatives of 19‐NAG, as well as of the other phase II metabolites of nandrolone, have never been tested before for linearity and their ability to quantify properly the target analytes. Though the TD2021NA implies the analysis of the combined free and glucuronide‐conjugated fraction as a matrix for the 19‐NA determination we did not include the free fraction in the assay, as the 19‐NA in the free fraction is negligible in comparison with its glucuronide conjugate.^33^ The two cut‐off concentration levels referred to in the TD2021NA for 19‐NA were used as thresholds for the rest of nandrolone phase II metabolites, namely the 19‐NAS, 19‐NEAS, 19‐NEG, and 19‐NES. It should be noted that though synthesized 19‐NEAS was included in the set of analytes to which the validation was performed, its quantification in real urine samples shall be considered indicative only, as a suitable reference material was not available and the synthesized standard is not an appropriate standard, under the provisions of ISO EN 17025.

For the quantitative confirmation, the dynamic range was tested in three different calibration curves of seven different calibration points (1, 2, 5, 20, 30, 60, and 100 ng mL^−1^) with four different replicates in each calibration point. The levels of quality control (QC) samples were established at 2.5 ng mL^−1^ and 15 ng mL^−1^, according to the two cut‐off levels, and were prepared in duplicates. Due to the presence of 19‐NAG in sub ng mL^−1^ concentrations in real urine samples, the three different calibration curves for the dynamic range assessment were prepared in artificial urine. Weighted calibration curves with a weighted factor of 1/x^2^ were constructed through IBM SPSS statistics tool 22. The limit of quantitation (LOQ) was defined as the lowest concentration level within the dynamic range at which its back‐calculated values are measured with precision (%RSD) and bias (% analytical error) lower than 15% and the S/N ratio higher than 10. Repeatability, intermediate precision, bias, and measurement uncertainty were determined by spiking 10 different real urine samples (two batches of five samples) in the two cut‐off levels on two different experimental days prepared by different analysts and analyzed in two different instruments. The same samples were analyzed again in several time intervals, the last one month later, in order to assess sample extract stability. The matrix effect was calculated for the same 10 spiked samples that were compared with a standard sample spiked without matrix (Milli‐Q water sample) in the same concentration levels. Additionally, bias for the analysis of 19‐NAG was evaluated through the analysis of a previously analyzed external quality assessment scheme (EQAS) sample distributed in previous time by WADA, available in the inventory of the Doping Control Laboratory of Athens (DCLA) and for which consensus value for the concentration of 19‐NA was available.

For the qualitative confirmation, selectivity was tested by analyzing 10 different real blank urine samples, in two batches of five samples each, from individuals of both sexes and varying in specific gravity and pH values. The limit of identification (LOI) was determined, by analyzing the same spiked samples used for the repeatability and intermediate precision determination, as the lowest concentration within the dynamic range in which all targets fulfill the identification criteria of TD2023IDCR.^16^ Robustness was assessed by using different instruments and different analysts on different days. Carryover was tested by the injection of a blank urine sample after the higher calibration point at 100 ng/ml.

### 2.5 Urine samples

#### 2.5.1 Real negative urine samples

Forty different real urine samples, twenty male and twenty female, with diverse pH and specific gravity values were used. These samples were available in the inventory of DCLA, previously analyzed and reported as negative for the presence of 19‐NA, and were used after being made anonymous.

#### 2.5.2 Excretion studies

Urine samples from two excretion studies involving the single‐dose oral administration of nandrolone decanoate (200 mg x 2 ml^−1^ injectable pharmaceutical solution) to two healthy male volunteers were used. Only samples that had previously screened by GC–MS/(MS) and have been diluted to be within the dynamic range (1 to 100 ng mL^−1^) of the developed method were used. The administration studies were approved by the Bioethics Committee of the National Center for Scientific Research “Demokritos” (Decision number: #92/2022). Information about these two excretion studies is following.

##### Excretion study 1

0.5 ml from 100 mg mL^−1^ vial of nandrolone decanoate. Urine samples were collected before administration (0 h) and then according to the time points: 0 h, 12 h, 24 h, 36 h, 48 h, 60 h, 72 h, 84 h, 120 h, 144 h, 168 h, 192 h, 216 h, and 240 h.

##### Excretion study 2

1 ml from 100 mg mL^−1^ vial of nandrolone decanoate. Urine samples were collected before administration (0 h) and then according to the time points: 0 h, 2 h, 4.5 h, 21.5 h, 30 h, 47 h, 64 h, 81 h, 120 h, 114 h, and 134 h.

## 3 RESULTS AND DISCUSSION

### 3.1 LC–MS chromatographic resolution

Sulfate metabolites of some anabolic androgenic steroids (AASs) are known to last for a longer period of time in urine samples in comparison with their glucuronic acid conjugates.^34^, ^35^, ^36^, ^37^, ^38^, ^39^, ^40^, ^41^, ^42^, ^43^ In the case of nandrolone, 19‐NAS has been proposed as a long‐term metabolite.^14^, ^15^, ^16^ However, due to the higher excreted amount of the glucurono‐conjugated 19‐NA, as well as the large amount of scientific evidence that has been accumulated over time concerning the response of this metabolite to various possible scenarios of its presence in athlete's urine samples, like endogenous presence,^44^, ^45^ pregnancy,^5^, ^6^ consumption of wild boar offal^7^, ^8^, ^9^, ^10^, ^11^ and androsterone demethylation,^12^ 19‐NAG is the metabolite of choice for the uncovering of nandrolone abuse.^33^ Other metabolites like 19‐NE in its glucuronide derivative are also important and when the ratio between 19‐NA/19‐NE is higher than 3, samples are reported as ATFs in the cases where GC‐C‐IRMS analysis is not conclusive.^3^ 19‐NEG is additionally important in cases where athletes use 5‐reductase inhibitors to mask nandrolone abuse.^46^

Despite its potential as a long‐term metabolite, 19‐NAS is currently ignored in the management of 19‐NA suspicious samples. This is mainly due to the laborious sample preparation needed for its analysis under the provisions of TD2023IDCR, as well as the conclusion drawn by Guay and coworkers from their study, in which the careful profiling of nandrolone metabolites between glucuronide and sulfate fraction does not permit a distinction between their synthetic or endogenous origin.^22^ Nevertheless, the conclusion of Guay et al is of major importance; however, it is worth pointing out that it is based only on the analysis of spotted samples obtained during sports drug testing, and therefore a more comprehensive investigation in this direction might be warranted.

We have recently presented that derivatization of intact phase II metabolites of oxo‐steroids with GRT enhances electrospray ionization (ESI) in positive ionization mode and simultaneously produces product ions of diagnostic value under CID conditions that enable the phase II metabolites identification under the provisions of the WADA TD2021IDCR.^29^ More recently, we developed a derivatization and shoot methodology for the confirmation of methenolone and mesterolone intact phase II metabolites in sub ng mL^−1^ concentration levels.^30^ Prompt from these results, a derivatization and shoot methodology for the identification of the phase II metabolites of nandrolone was developed and presented herein. The developed method is simple, involving only GRT derivatization of intact urine aliquots, that have been fortified with ISTDs, and direct analysis in a LC–MS/(HRMS) system, and uses a very low amount of sample, 100 μl per aliquot. The application of this methodology in the case of confirmation of 19‐NA findings in the frame of doping control permits the detailed analysis of the oxo‐metabolic profile of nandrolone or its precursors in a single run with both convenient and effective way, broads' analytical possibilities, and might be proved useful in the future for the management of nandrolone cases.

**FIGURE 1:**
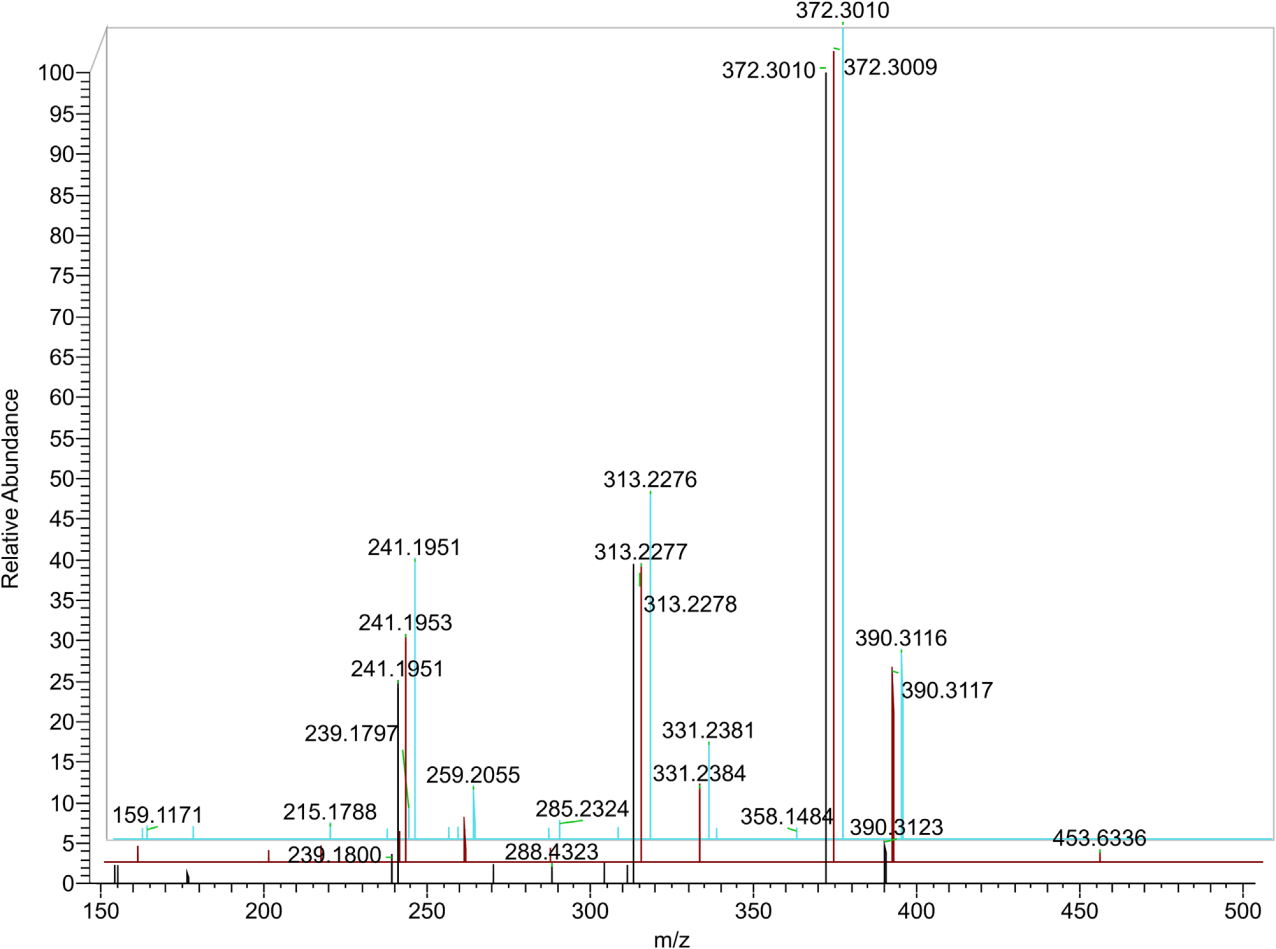
Product ion spectra of 19‐NEAS (blue), 19‐NAS (red), and 19‐NES (black).

**FIGURE 2:**
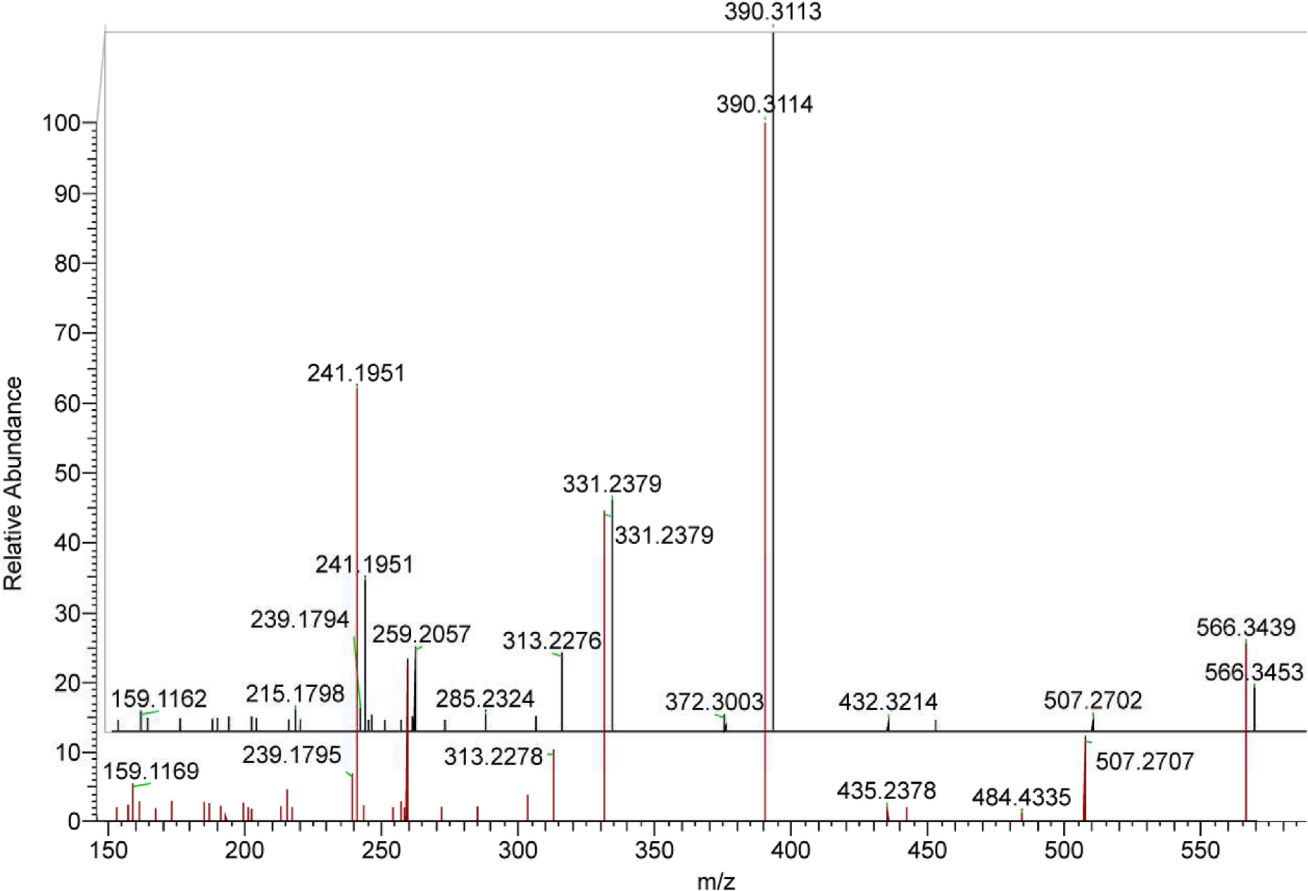
Product ion spectra of 19‐NAG (red) and 19‐NEG (black).

GRT derivatives of 19‐NA, 19‐NE, 19‐NEA, and 19‐NEE in their sulfate conjugate as well as 19‐NA and 19‐NE in their glucuronide form presented abundant molecular ions under positive ESI ionization with *m/z* 470.2689 and 566.3441 respectively. 19‐norepietiocholanolone sulfate (19‐NEES), though synthesized and preliminary analyzed, was excluded from the targeted metabolites, as an initial analysis of nandrolone post‐administration samples proved that this analyte is not a nandrolone metabolite. 19‐NAS, 19‐NES, and 19‐NEAS exhibit similar product ion spectra as presented in Figure 1. Only minor differences regarding the product ion ratios were obtained, with major product ions resulting from M^+^‐SO_3_; 390.3121, M^+^‐SO_3_‐H_2_O; 372.3015, M^+^‐SO_3_‐Me_3_N; 331.2386, M^+^‐SO_3_‐H_2_O‐Me_3_N; 313.2280, M^+^‐SO_3_‐GRT; 259.2062 and M^+^‐SO_3_‐H_2_O‐GRT; 241.1956. The same pattern has arisen for the GRT derivatives of 19‐NAG and 19‐NEG with differences in product ion ratios being more characteristic. Major product ions are resulting from M^+^‐glucuronic acid; 390.3121, M^+^‐glucuronic acid‐Me_3_N; 331.2386, M^+^‐glucuronic acid‐H_2_O‐Me_3_N; 313.2280, M^+^‐glucuronic acid‐GRT; 259.2062, M^+^‐glucuronic acid‐H_2_O‐GRT; 241.1956, M^+^‐Me_3_N; 507.2705 and M^+^‐glucuronic acid‐H_2_O; 372.3015 only for 19‐NEG. Product ion spectra of 19‐NAG and 19‐NEG are presented in Figure 2. The retention times, product ions, chemical formulas, and abundance information for all the analytes of interest are presented in Table 1.

**TABLE 1:** Retention times, product ions, chemical formulas, and abundance information for all the analytes of interest.

Analyte	RT (min)	Precursor ion/formula	Product ion 1/formula/abundance	Product ion 2/formula/abundance	Product ion 3/formula/abundance	Product ion 4/formula/abundance	Product ion 5/formula/abundance	Product ion 6/formula/abundance	Product ion 7/formula/abundance
**19‐NEAS**	14.83	470.2689/	390.3121/	372.3015/	331.2386/	313.2280/	259.2062/	241.1956/	‐
		C_23_H_40_N_3_O_5_S	C_23_H_40_N_3_O_2_/	C_23_H_38_N_3_O_1_/	C_20_H_31_N_2_O_2_/	C_20_H_29_N_2_O_1_/	C_18_H_27_O_1_/	C_18_H_25_/	
			25%	100%	47%	42%	6%	33%	
**19‐NAS**	23.15	470.2689/	390.3121/	372.3015/	331.2386/	313.2280/	259.2062/	241.1956/	‐
		C_23_H_40_N_3_O_5_S	C_23_H_40_N_3_O_2_/	C_23_H_38_N_3_O_1_/	C_20_H_31_N_2_O_2_/	C_20_H_29_N_2_O_1_/	C_18_H_27_O_1_/	C_18_H_25_/	
			23%	100%	9.5%	38%	4%	30%	
**19‐NES**	25.59	470.2689/	390.3121/	372.3015/	331.2386/	313.2280/	‐	241.1956/	‐
		C_23_H_40_N_3_O_5_S	C_23_H_40_N_3_O_2_/	C_23_H_38_N_3_O_1_/	C_20_H_31_N_2_O_2_/	C_20_H_29_N_2_O_1_/		C_18_H_25_/	
			3%	100%	2%	32%		25%	
**19‐NEG**	35.30	566.3441/	390.3121/	372.3015/	331.2386/	313.2280/	259.2062/	241.1956/	507.2705/
		C_29_H_48_N_3_O_8_	C_23_H_40_N_3_O_2_/	C_23_H_38_N_3_O_1_/	C_20_H_31_N_2_O_2_/	C_20_H_29_N_2_O_1_/	C_18_H_27_O_1_/	C_18_H_25_/	C_29_H_48_N_3_O_8_/
			100%	1.2%	35%	10%	10%	25%	1.4%
**19‐NAG**	36.87	566.3441/	390.3121/		331.2386/	313.2280/	259.2062/	241.1956/	507.2705/
		C_29_H_48_N_3_O_8_	C_23_H_40_N_3_O_2_/	‐	C_20_H_31_N_2_O_2_/	C_20_H_29_N_2_O_1_/	C_18_H_27_O_1_/	C_18_H_25_/	C_29_H_48_N_3_O_8_/
			100%		47%	8%	22%	67%	11%

**FIGURE 3:**
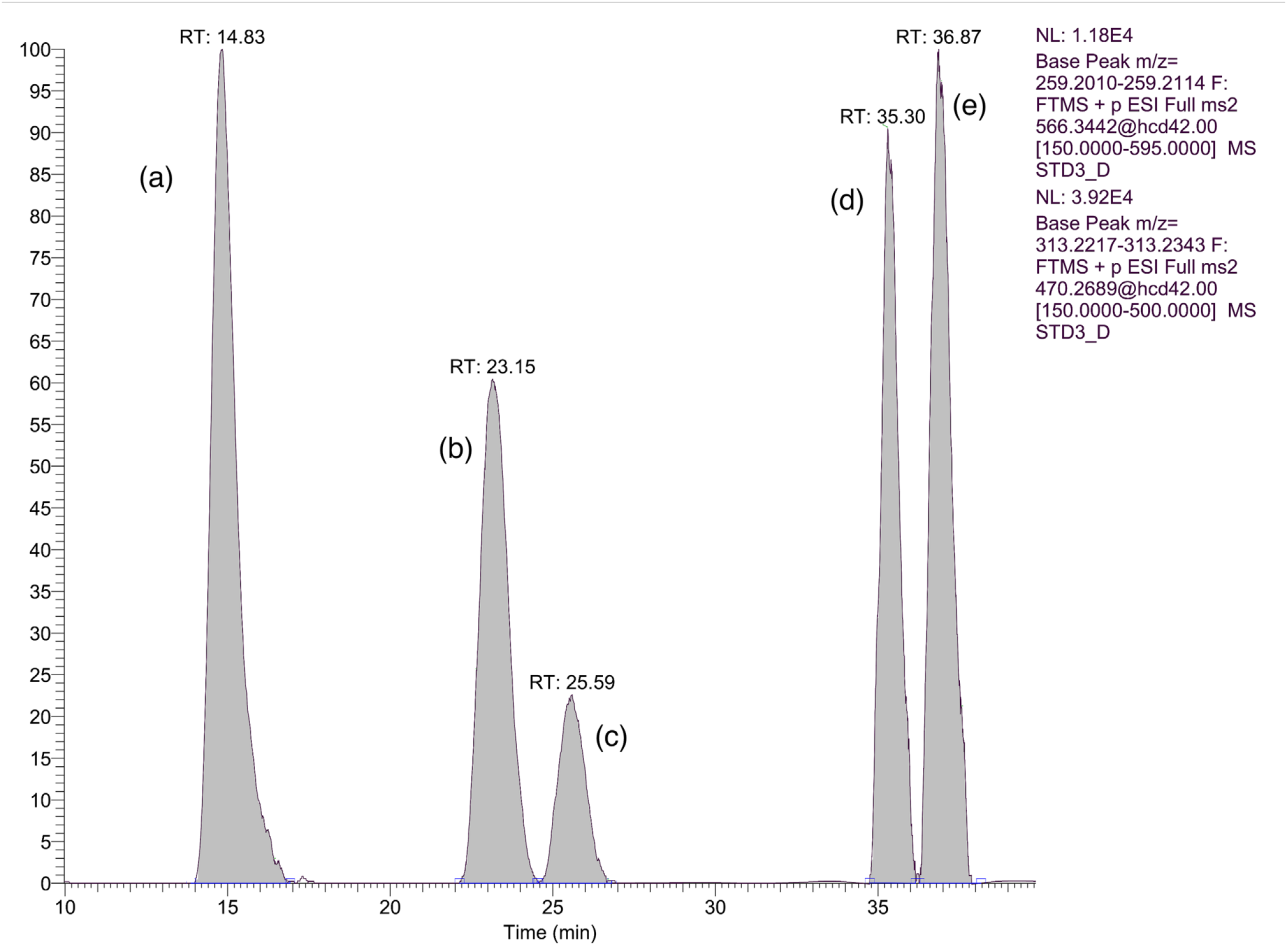
Merged extracted ion chromatogram of the transition 470.2689 > 313.2280 for the analytes a to C and transition 566.3441 > 259.2062 for analytes D and E that corresponding to: A) 19‐NEAS, B) 19‐NAS, C) 19‐NES D) 19‐NAG, and E) 19‐NEG.

As 19‐NA, 19‐NE, and 19‐NEA in their sulfate conjugates, as well as 19‐NA and 19‐NE in their glucuronide conjugates, exhibited marginal differences in their product ion spectra, it was important to achieve adequate chromatographic separation. These compounds are isomeric with minor variations in their stereochemistry and hence minor differences in their polarities, which are further altered by the introduction of the charged GRT derivative in their molecule. Consequently, liquid chromatographic separation constituted an analytical challenge, especially for the separation of the pair 19‐NAG and 19‐NEG as GRT derivatives. Extensive experimentation was conducted to accomplish this chromatographic separation as we could not reproduce our previously published results in reference [29], where the two metabolites were separated using a Zorbax eclipsed C18 column. New batches of this column were extensively tested, along with other various C18 and C8 columns from different brands and with different sorbents. The efficient separation of 19‐NAG and 19‐NEG, as well as 19‐NAS, 19‐NES, and 19‐NEAS, was achieved using a series combination of Thermo Hypersil Green PAH phenyl column with an Agilent poroshil C18 column, along with a long chromatographic gradient. The final chromatographic run is 55 min and successfully achieves efficient separation of nandrolone sulfate and glucuronide oxo‐metabolites in a single run. Although the chromatographic run is considered long, it is equal to or even less than that presented in previously published methods where the chromatographic resolution of the same phase II nandrolone's metabolites pairs was performed without derivatization.^23^, ^24^ Additionally, after comparing the lengthy and laborious sample preparation described in the above‐mentioned two publications in comparison with the dilute and shoot approach developed herein, our methodology is simple and cost‐effective and has an advantage that none of the previous liquid chromatography–mass spectrometry (LC–MS) approaches have, it fulfills the requirements of TD2023IDCR. The chromatographic separation is presented in Figure 3.

### 3.2 Validation results

Regarding quantitative confirmation, after the evaluation of the calibration curves analysis, the assay proved linear in the range of 1–100 ng mL^−1,^ and the LOQ was determined to be 1 ng mL^−1^ for all the analytes of interest. Calibration curves were tested in both real urines and artificial urine. Artificial urine was preferred, due to the endogenous presence of 19‐NAG in amounts lower than 1 ng mL^−1^ in real urine spiked samples, which resulted in non‐acceptable analytical errors in the back calculated concentration values in the lower levels of the calibration curves. The issue of quantification of endogenous compounds (like 19‐NA) in the absence of a real blank matrix has been discussed thoroughly in the literature and the choice of the surrogate matrix is considered an appropriate alternative.^47^, ^48^ Repeatability and intermediate precision were both found to be lower than 10% for all the analytes for both 2.5 and 15 ng mL^−1^ concentration levels. Both bias and uncertainty were found to be lower than 15% in all cases for both 2.5 and 15 ng mL^−1^ hypothetical threshold, except for 19‐NEAS where an arbitrary U_ref_ of 20% was used due to the absence of proper reference material. The evaluation of the bias by the reanalysis of the previously analyzed EQAS sample resulted that there is no evidence of any unaccounted bias. The sample extracts proved to be stable for at least one month. Additionally, mean matrix effects are expressed in %, where a positive value indicates ion enhancement and negative values ion suppression, and were found 4.38 ± 34.2% for 19‐NAG, 15.1 ± 41.9% for 19‐NAS, 17.7 ± 41.3% for 19‐NEAS, 0.86 ± 52.1% for 19‐NEG, and 14.9 ± 44.7% for 19‐NES. Quantitative validation results are presented in Table 2 for both 2.5 and 15 ng mL^−1^.

**TABLE 2:** Quantitative method validation results for both 2.5 and 15 ng mL^−1^.

Validated parameter	Repeatability %		Intermidiate precision %		Bias %		Uncertainty %	
**cut off (ng/mL** ^**−1**^ **)**	**2,5**	**15**	**2,5**	**15**	**2,5**	**15**	**2,5**	**15**
**19‐NAG**	5,46	4,18	5,71	4,18	8,47	5,14	8,66	5,31
**19‐NAS**	7,97	1,33	8,37	1,33	11,0	4,57	11,4	4,59
**19‐NEAS**	6,08	6,91	6,32	6,92	20,8	20,3	20,9	20,4
**19‐NEG**	8,33	4,12	8,40	4,12	6,33	3,93	6,86	4,14
**19‐NES**	6,19	5,63	6,56	5,66	8,25	12,3	8,51	12,5

As for qualitative confirmation, the selectivity was 100% as none of the analyzed samples was observed to signal for any analyte of interest in concentrations higher than 2.5 ng mL^−1^. Indeed, only signals for the 19‐NAG were observed at sub‐ng mL^−1^ levels, while the other analytes were detected at a very low concentration. The LOI was established at 2.5 ng mL^−1^ for all analytes. Given that this is a confirmation method, this concentration is considered adequate, according to the cut‐off value of 2.5 ng mL^−1^ posed by TD2021NA, and lower concentrations were not tested. The assay was proven to be robust as the determinations of the analytes of interest were independent of the analyst, the experimental day, and the instrument used for these determinations. Carryover was not observed. Overall, the developed method is capable of quantifying and identifying all known phase II oxo‐metabolites of nandrolone, it is fit for purpose and offers the advantages of minimal sample consumption, convenient sample preparation, and orthogonality to the traditional GC–MS/(MS) method. Taking into account literature reports where a) the excretion of 19‐NAG is faster than that of sulfate metabolites after nandrolone prohormone application,^22^ b) 19‐NAS is the long‐term marker of nandrolone preparations abuse^14^, ^15^, ^16^ c) the prevalence of 19‐NAG in cases where the presence of nandrolone metabolites in athlete's urine is reasonably justified like pregnancy and consumption of wild boar offal,^22^ and d) the prevalence of 19‐NA findings with endogenous isotopic signature,^49^ the implementation of the method presented herein may increase metabolic certainty and help the result management of nandrolone cases with low levels of 19‐NAG.

### 3.3 Analysis of real samples

Two sets of real urine samples were analyzed by the validated method. The first set consisted of the real negative urine samples and was analyzed in order to evaluate further the selectivity of the method and check for any unexpected findings concerning the phase II metabolites of nandrolone. The second set of samples involved the post‐administration samples of the two excretion studies, where the values of nandrolone phase II oxo‐metabolites were estimated. The concentrations of 19‐NAG and 19‐NEG in the second set of samples were compared with the concentration values of their deliberated free counterparts obtained by analysis with the traditional GC–MS/(MS) method. In the first set of samples, all samples were found to be negative for 19‐NAG at levels higher than 2.5 ng mL^−1^. Furthermore, while 19‐NAG was found in amounts lower than 1 ng mL^−1^, other phase II metabolites, including 19‐NAS, were less significant or undetectable. Regarding the second set of samples, the results for 19‐NAG and 19‐NEG were compared with those obtained using traditional GC–MS/(MS) analysis after hydrolysis of the glucuronide fraction, extraction and derivatization. A comparison of the concentrations obtained for 19‐NAG and 19‐NEG was made by regression analysis of the values obtained by the two different methods, resulting in a linear response with R^2^ > 0.98. Additionally, the pair T‐test confirmed that the assumption of a zero difference between pairs of measurements for 19‐NAG and 19‐NEG obtained from the two different analytical methods was correct and proves that the new method is equally valid for the estimation of 19‐NA concentration within the linearity range. Furthermore, all the expected metabolites were found to be present. It should be noted that although the detailed investigation of the excretion profile of nandrolone phase II metabolites was not the primary purpose of this analysis, the samples from one excretion study presented a relatively high amount of 19‐NES, while in the second excretion study, this metabolite was not excreted at all, evidencing an inter‐individual variation in the excretion of this metabolite. More specifically, 19‐NAG was the dominant metabolite with concentrations ranging from 468 to 28 ng mL^−1^, followed by 19‐NEG with concentrations ranging from 308 to 14 ng mL^−1^, 19‐NAS from 168 to 1.1 ng mL^−1^, 19 NEAS from 99 to 1.5 ng mL^−1^, and finally 19‐NES from 7 to sub‐ ng mL^−1^. Values that were out of the linearity range were estimated after proper dilution of the corresponding samples. Finally, the method was applied to the previously analyzed WADA EQAS sample containing 19‐NA with a known value and the obtained z score was found to be 0.54.

**FIGURE 4:**
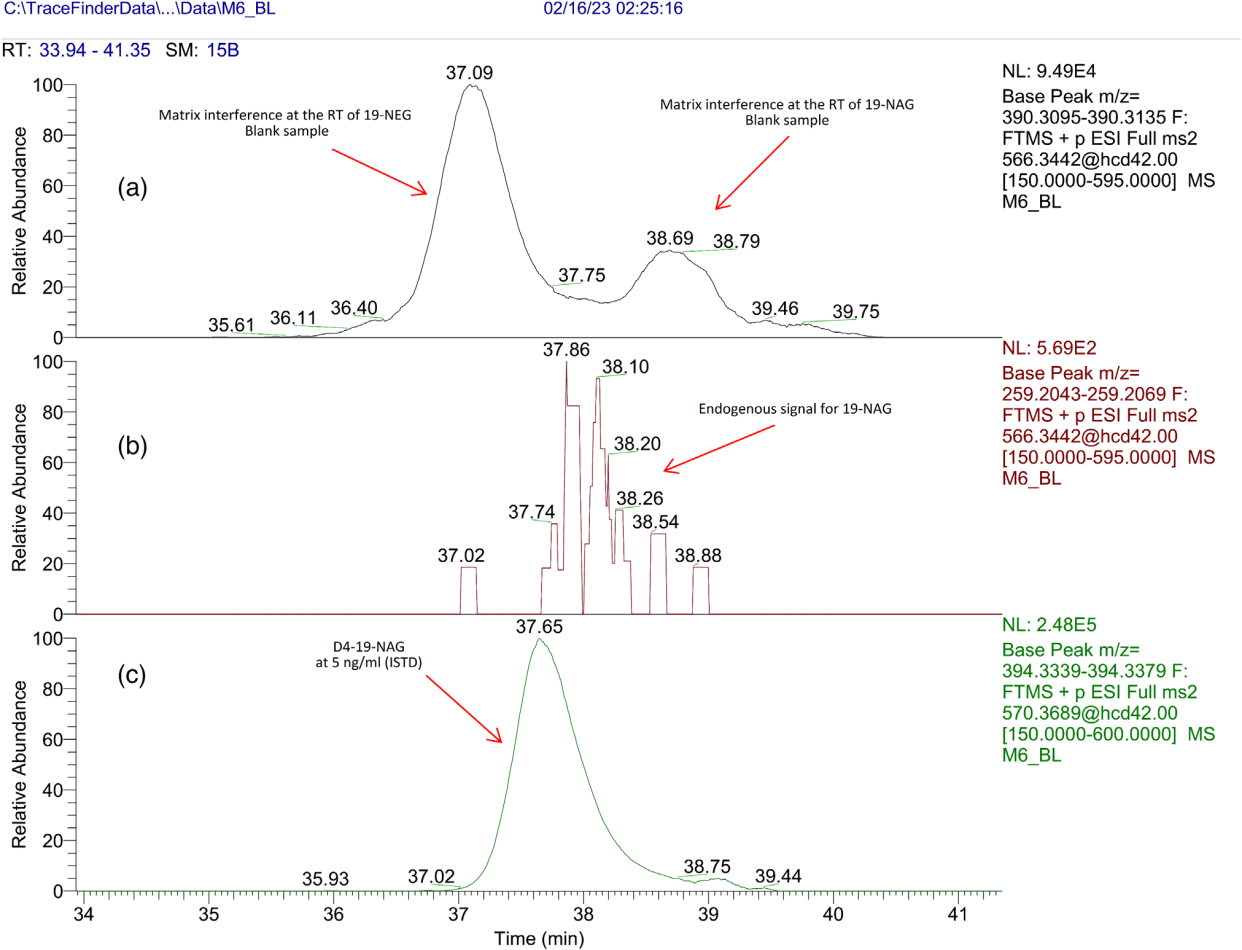
The most intense matrix interference obtained during the course of this study. A) Ion transition of 566.3441 > 390.3121 of a blank sample B) extracted ion transition of 566.3441 > 259.2062, C) extracted ion transition of ISTD D4–19‐NAG.

**FIGURE 5:**
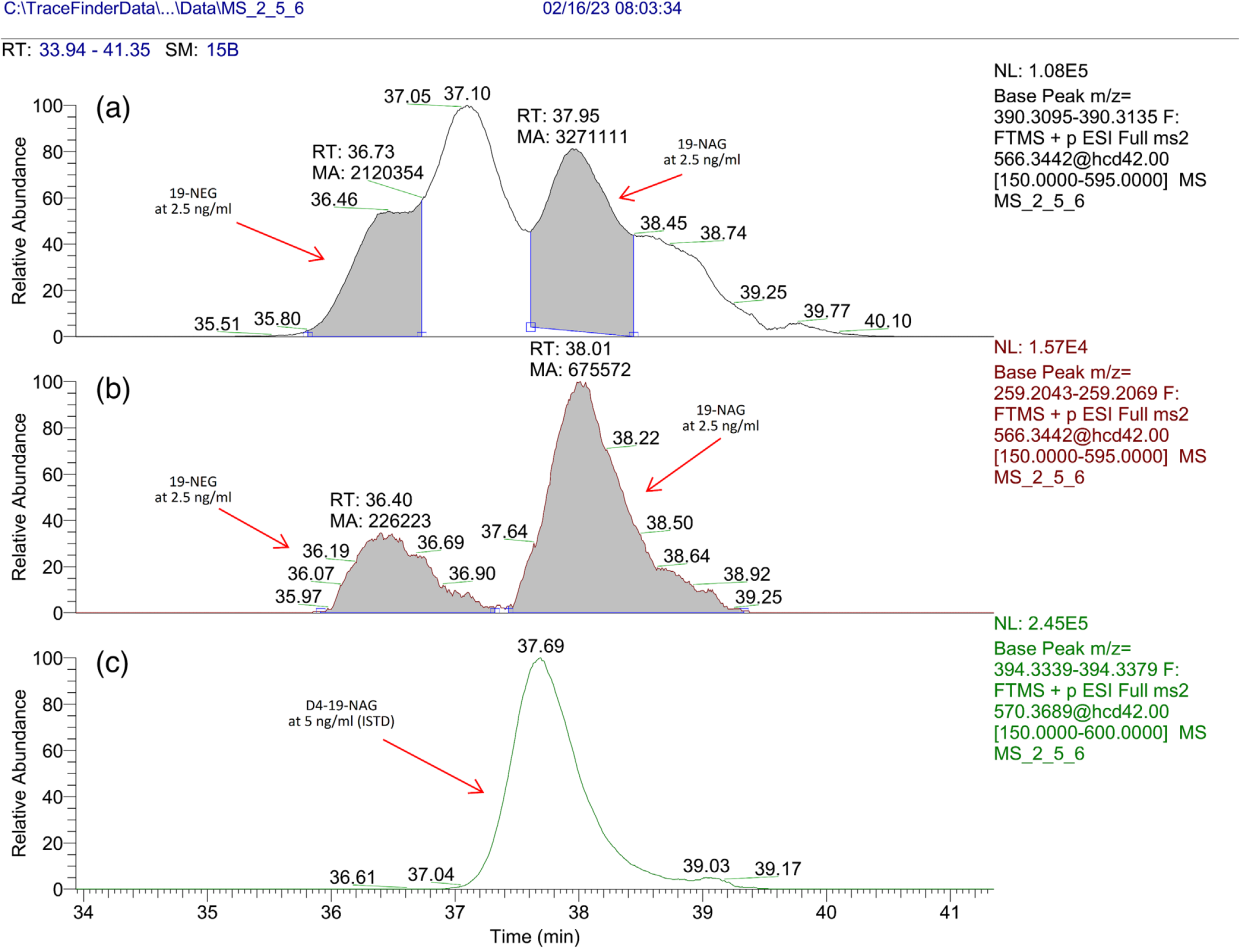
The most intense matrix interference obtained during the course of this study. A) Ion transition of 566.3441 > 390.3121 of the same blank sample spiked with 2.5 ng mL^−1^ of 19‐NEG and 19‐NAG, B) extracted ion transition of 566.3441 > 259.2062, C) extracted ion transition of ISTD D4–19‐NAG.

It is important to note that in eight out of the 40 different real authentic urine samples used for the evaluation of the selectivity of the assay, a matrix interference was observed, which resulted in the poor separation of 19‐NAG and 19‐NEG from matrix at the concentration level of the endogenous 19‐NAG for the major ion transitions 566.3441 > 390.3121 and 566.3441 > 331.2386 with an extracted mass window of 10 ppm. The most intense matrix interference was observed after the analysis of a male volunteer sample and is presented in Figure 4 (blank sample) and Figure 5 (the same blank sample spiked with 19‐NAG and 19‐NEG at 2.5 ng mL^−1^).

This matrix interference was present regardless of the mass resolution (17,5, 35, or 70 K) or the mass extraction window (10 or 5 ppm) and is of unknown origin. Furthermore, after careful inspection of its product ion spectrum, we observe all ions found in the product ion spectra of 19‐NAG and 19‐NEG found in pure solutions except from the ion with *m/z* 259.2059. Additionally, two minor product ions with *m/z* 254.1902 and 296.2013 that are not present in any of the nandrolone metabolites can be found for interference. Hence, ion transition 566.3441 > 259.2062 is used for the discrimination of 19‐NAG from the interference and although it is the ion transition with the lowest abundant, it was chosen as the quantitative trace. Additionally, minor interferences were also obtained for the traces of 19‐NAS and 19‐NES but again the ion transitions 470.2689 > 259.2062 and 470.2689 > 241.1956 improve the analysis of the metabolites of interest.

## 4 CONCLUSION

A derivatization and shoot method were developed for the first time for the simultaneous quantification and identification of nandrolone phase II oxo‐metabolites. The assay was based on intact urine aliquots that were diluted and at the same time derivatized with GRT aquatic solution and analyzed without any further purification in LC–MS/(HRMS). The developed method was able to quantify 19‐NAG and 19‐NEG, as well as 19‐NAS, 19‐NES, and 19‐NEAS in athletes' urine samples in one run. The presented methodology permits the monitoring of the comprehensive metabolic profile of nandrolone phase II oxo‐metabolites, it is orthogonal to the traditional GC–MS/(MS) methods and requires a minimum sample volume (100 μl). Full quantitative and qualitative validation of the assay was performed and the validated method was applied to real urine samples. The utilization of this methodology for confirming 19‐NA findings in doping control is straightforward, cost‐effective, expands the range of analytical possibilities, and holds potential for future applications in the management of nandrolone cases, improving the metabolic certainty in each 19‐NA finding.

## AUTHOR CONTRIBUTIONS

P.S.: investigation, formal analysis, data curation, validation, visualization, writing—original draft, and writing—review/editing. P.K.: formal analysis, data curation, writing—review/editing. M. P.: resources, and writing—review/editing. Y.S.A.: conceptualization, methodology, investigation, data curation, validation, visualization, funding acquisition, project administration, supervision, writing—original draft, and writing—review/editing.
